# A-Disintegrin and Metalloproteinase (ADAM) 17 Enzymatically Degrades Interferon-gamma

**DOI:** 10.1038/srep32259

**Published:** 2016-08-30

**Authors:** Hiroyuki Kanzaki, Fumiaki Shinohara, Maiko Suzuki, Satoshi Wada, Yutaka Miyamoto, Yuuki Yamaguchi, Yuta Katsumata, Seicho Makihira, Toshi Kawai, Martin A. Taubman, Yoshiki Nakamura

**Affiliations:** 1 Department of Orthodontics, School of Dental Medicine, Tsurumi University, 2-1-3 Tsurumi, Tsurumi-ku, Yokohama, Kanagawa pref., 230-8501, Japan; 2 Tohoku University Hospital, Maxillo-Oral Disorders, 4-1 Seiryo-machi, Aoba-ku, Sendai, Miyagi pref. 980-8575, Japan; 3 Tohoku University Graduate School of Dentistry, Oral Microbiology, 4-1 Seiryo-machi, Aoba-ku, Sendai, Miyagi pref. 980-8575, Japan; 4 The Forsyth Institute, Department of Immunology and Infectious Diseases, 245 First Street, Cambridge, MA, 02142, USA; 5 Department Mineralized Tissue Biology, 245 First Street, Cambridge, MA 02142, USA; 6 Section of Fixed Prosthodontics, Division of Oral Rehabilitation, Faculty of Dental Science, Kyushu University, 3-1-1 Maidashi Higashi-ku, Fukuoka 812-8582, Japan; 7 Harvard School of Dental Medicine, Department of Oral Medicine, Infection, and Immunity, Boston, MA 02115, USA; 8 Harvard School of Dental Medicine, Department of Developmental Biology, Boston, MA 02115, USA

## Abstract

Interferon-gamma (IFN-γ) is a pleiotropic cytokine that exerts anti-tumor and anti-osteoclastogenic effects. Although transcriptional and post-transcriptional regulation of IFN-γ is well understood, subsequent modifications of secreted IFN-γ are not fully elucidated. Previous research indicates that some cancer cells escape immune surveillance and metastasize into bone tissue by inducing osteoclastic bone resorption. Peptidases of the a-disintegrin and metalloproteinase (ADAM) family are implicated in cancer cell proliferation and tumor progression. We hypothesized that the ADAM enzymes expressed by cancer cells degrades IFN-γ and attenuates IFN-γ-mediated anti-tumorigenic and anti-osteoclastogenic effects. Recombinant ADAM17 degraded IFN-γ into small fragments. The addition of ADAM17 to the culture supernatant of stimulated mouse splenocytes decreased IFN-γ concentration. However, ADAM17 inhibition in the stimulated mouse T-cells prevented IFN-γ degradation. ADAM17-expressing human breast cancer cell lines MCF-7 and MDA-MB-453 also degraded recombinant IFN-γ, but this was attenuated by ADAM17 inhibition. Degraded IFN-γ lost the functionality including the inhibititory effect on osteoclastogenesis. This is the first study to demonstrate the extracellular proteolytic degradation of IFN-γ by ADAM17. These results suggest that ADAM17-mediated degradation of IFN-γ may block the anti-tumorigenic and anti-osteoclastogenic effects of IFN-γ. ADAM17 inhibition may be useful for the treatment of attenuated cancer immune surveillance and/or bone metastases.

Interferon gamma (IFN-γ) is a cytokine that exerts an anti-tumor effect through the activation of natural killer cell surveillance^1^,^2^,^3^,^4^. Moreover, IFN-γ has been proposed as a prognostic factor in cancer therapy^5^,^6^,^7^,^8^. In addition to its anti-tumor functions, IFN-γ also exerts an anti-osteoclastogenic effect by inducing degradation of tumor necrosis factor receptor-associated factor 6 (TRAF6), resulting in the inhibition of receptor activator of nuclear factor (NF)-kappa B ligand (RANKL) signaling^9^.

The IFN-γ expression is controlled via transcriptional regulatory mechanisms consisting of transcriptional factors, such as NF-kappa B, Smad, and STAT^10^, in addition to post-transcriptional modifications involving, for example, RNA processing and alternative splicing^11^. However, while the regulation of IFN-γ secretion has been studied^12^,^13^, both IFN-γ secretory processes and their subsequent post-translational modification have, for the most part, been neglected, except for internalization and degradation of IFN-γ after receptor binding^14^,^15^.

Many studies have shown that some cancer cells can escape immune surveillance^16^,^17^,^18^,^19^, which is mediated in part by immune cell derived anti-cancer factor, IFN-γ^4^,^20^,^21^,^22^, indicating that cancer cells can attenuate immune surveillance via the inactivation of secreted IFN-γ. In addition, certain cancer cells metastasize into bone tissue and induce osteoclastic bone resorption^23^,^24^,^25^, indicating that the cancer cells might promote the suppression of the anti-osteoclastogenic activity of the host.

Proteinases of the a-disintegrin and metalloproteinase (ADAM) family have been implicated in tumorigenesis and cancer spread^26^,^27^,^28^. Furthermore, studies have demonstrated a significant correlation between the expression of ADAM enzymes and tumor stage progression^29^,^30^,^31^,^32^,^33^. These data led to the hypothesis that cancer cells may block anti-tumor cell defenses and neutralize the anti-osteoclastogenic effects through the expression of ADAM proteinases. We hypothesized that some ADAMs expressed by cancer cells could degrade IFN-γ-attenuated or IFN-γ-mediated anti-tumorigenic and anti-osteoclastogenic effects. To test this hypothesis, we performed *in vitro* experiments using primary lymphocytes and cell lines, as well as recombinant IFN-γ and ADAM enzymes.

## Results

### Recombinant ADAM17 (rADAM17), but not ADAM10, degrades recombinant IFN-γ

**Figure 1:**
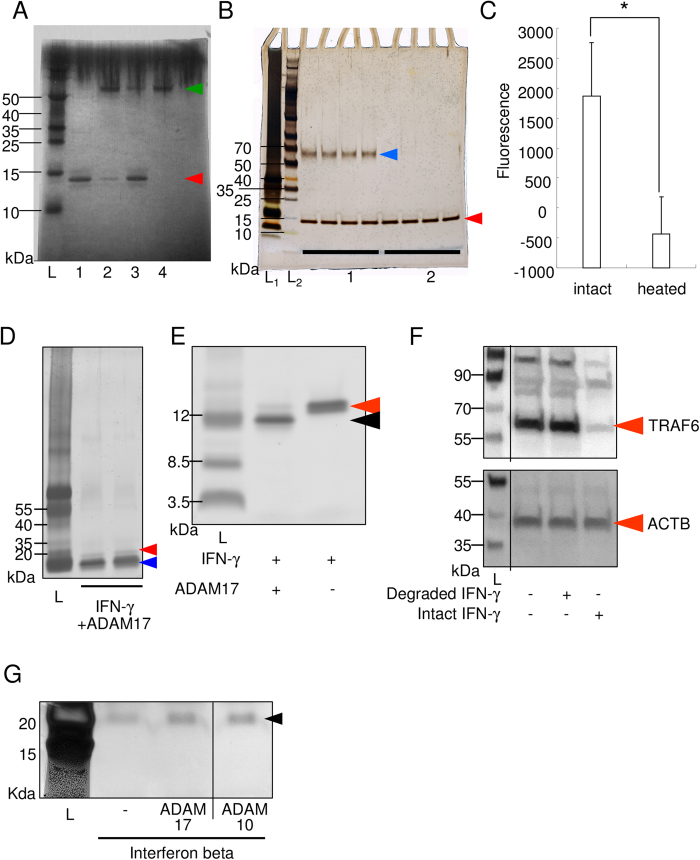
ADAM17, but not ADAM10, degrades IFN-γ. (**A**) ADAM17 reaction with IFN-γ (Coomassie blue staining of the PAGE gel). The green arrowhead indicates the size of recombinant ADAM17 (rADAM17) (52 kDa), and the red arrowhead indicates the intact recombinant IFN-γ (rIFN-γ) (15.6 kDa). L, molecular weight marker; 1, rIFN-γ; 2, rIFN-γ+rADAM17; 3, rIFN-γ+heat-inactivated rADAM17; 4, rADAM17. A representative photograph is shown. The size of molecular weight marker is indicated on the left side. (**B**) ADAM10 reaction with IFN-γ (Coomassie blue staining). The blue arrowhead indicates the size of the recombinant ADAM10 (rADAM10: 60kDa), and the red arrowhead indicates recombinant IFN-γ (rIFN-γ). 1, rIFN-γ+rADAM10; 2, rIFN-γ (n = 4 each). (**C**) Measurement of functional activity of recombinant ADAM10 using fluorescent substrate. Intact or heat inactivated ADAM10 (100 ng) and fluorescent substrate (ES-010; R&D systems: 5 nmol) were incubated at 37 °C and fluorescence was measured at excitation 320 nm and emission 405 nm. *p < 0.05 between the groups. (**D**) Silver staining of the PAGE gel. The red arrowhead indicates intact recombinant human IFN-γ (hIFN-γ) (15.6 kDa), and blue arrowhead indicates degraded small fragments of hIFN-γ. The fragment indicated by the blue arrowhead was excised and subjected to nano LC-MS/MS analysis. (**E**) Silver staining of the PAGE gel. IFN-γ with and without ADAM17 were incubated for the same period, and electrophoresed. Red arrowhead indicates intact IFN-γ and the black arrowhead indicates degraded fragment. (**F**) Functional assay of degraded IFN-γ fragment using western blot analysis for TRAF6 and loading control, ACTB. Separate lanes from the same blot had been spliced together, and lines indicate the places at which the lanes were joined. (**G**) IFN beta was not degraded by ADAM10 or ADAM17. Recombinant human IFN beta (300 ng) was incubated with or without recombinant ADAM10 or ADAM17 (100 ng) in reaction buffer for 2 hours. Then samples were reduced, denatured, and electrophoresed in TGX precast gel, and silver staining was performed. Arrowhead indicates the size of intact recombinant human IFN beta (22.5 kDa). Separate lanes from the same gel had been spliced together, and lines indicate the places at which the lanes were joined.

The correlation between ADAM17 expression and tumor progression in breast cancer was reported^29^. Therefore, we hypothesized that ADAM17 could promote tumorigenesis by its proteolytic degradation of the anti-proliferative cytokine IFN-γ. We first tested whether ADAM17 could degrade IFN-γ using recombinant proteins. Figure 1A shows a decrease in the density of the IFN-γ band after incubation with rADAM17 (lane 2) compared to control, intact IFN-γ sample (lane 1). This decrease in IFN-γ caused by ADAM17 was reversed by heat inactivation of rADAM17, indicating that ADAM17 enzymatically degraded IFN-γ. On the other hand, the incubation of IFN-γ with ADAM10 did not cause any changes in the density of the IFN-γ band (Fig. 1B). To eliminate the possibility of recombinant ADAM10 has no enzymatic activity, we measured enzymatic activity using fluorescent substrate (Fig. 1C). Recombinant ADAM10 has certain enzymatic activity. These data were in support with the enzymatic degradation of IFN-γ by ADAM17 over ADAM10.

### ADAM17 degrades IFN-γ into small fragments

We then examined whether ADAM17 exerted exo- or endopeptidase activity against IFN-γ. Silver staining revealed that the treatment with ADAM17 resulted in small-sized bands located below the intact IFN-γ band (Fig. 1D). Mass spectrometry of the excised smallest band, as indicated by the blue arrowhead in Fig. 1D, revealed that the amino acid sequence of this band corresponded to the proximal C-terminal region of IFN-γ (data not shown). These data indicate that ADAM17 demonstrates endopeptidase activity towards IFN-γ. To eliminate the possibility of self-degradation of IFN-γ, IFN-γ without ADAM17 was incubated for the same period, electrophoresed and stained with silver stain kit (Fig. 1E). There ware no small sized fragment bands of IFN-γ around 10kDa, signifying the enzymatic digestion by ADAM17 and not self-degradation of IFN-γ.

To analyze whether the degraded small fragments of IFN-γ still have functional activity, TRAF6 signaling after RANKL stimulation was observed using RAW 264.7 cells, because it was reported that RANKL stimulation give rise the induction of TRAF6 signaling, and IFN-γ induces degradation of TRAF6^9^. As shown in Fig. 1F, intact IFN-γ strikingly attenuated TRAF6 signaling, though degraded IFN-γ fragment showed no effect. This data suggests that degraded IFN-γ can lose its functional activity.

### IFN-β was not degraded by ADAM17 or ADAM10

The interferon family contains both IFN-γ and IFN-β^34^. Therefore, we asked if ADAM17 and ADAM10 could also degrade IFN-β. Figure 1G showed no observable difference between the lane of IFN-β alone and the lane of IFN-β reacted with ADAM17 and ADAM10, indicating that neither ADAM could degrade IFN-β.

### Neutralizing activity of the anti-ADAM17 antibody

We selected nine potentially immunogenic epitopes to explore which part of the ADAM17 amino acid sequence was suitable for generation of a neutralizing antibody. The corresponding synthetic peptides were used to raise polyclonal antibodies, followed by a comparison of their neutralizing activity against ADAM17. As seen in Supplementary Figure 1A, the affinity-purified polyclonal antibodies against peptide Nos 4 and 9 significantly reduced the enzymatic activity of ADAM17. Interestingly, the affinity-purified polyclonal antibody against peptide No. 5 showed weak inhibition, even though the region was proximal to the peptidase catalytic domain. In addition, the affinity-purified polyclonal antibody against peptide No. 7 showed faint inhibition in spite of the epitope being comprised of the catalytic center of ADAM17.

We then established a hybridoma line that produced the mAb against peptide No. 9, performed an epitope mapping, and compared the antibody neutralizing activity to that of an ADAM family inhibitor, TAPI2. Supplementary Figures 2B,C show the results of the epitope mapping. Pre-incubation of the mAb with peptide 9B resulted in a significant reduction of antibody reactivity against rADAM17. Similarly, the binding of the mAb to peptide 9B was higher than its binding to the other peptides. These data indicate that the epitope sequence of the established mAb is YPIAVSGD (UniProt P78536: 436-443), which is specific for ADAM17, based on the negative BLAST search for cross-reactive proteins (http://blast.ncbi.nlm.nih.gov/Blast.cgi) (data not shown). Supplementary Figure 2D showed the neutralizing ability of rADAM17 and 50% neutralizing doses (ND_50_) for the mAb and TAPI2 inhibitor, which were approximately 10^3^ and 10^6^ pM, respectively. These results suggest that the mAb obtained against ADAM17 is specific and effective in blocking the enzymatic activity of ADAM17.

**Figure 2:**
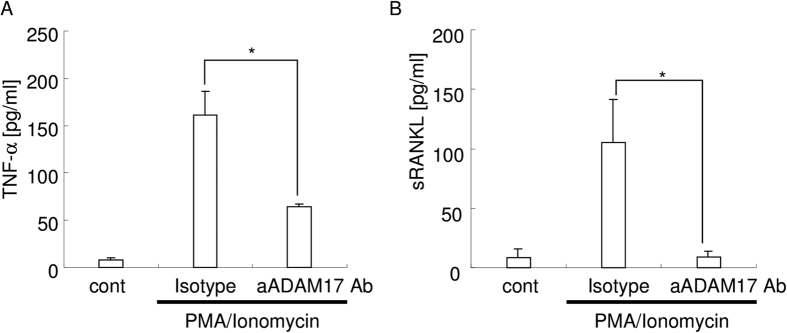
Anti-ADAM17 monoclonal antibody. (**A**) Inhibition activity of the anti-ADAM17 mAb (100 ng/mL) against TNF-α release. *p < 0.05 between the groups. (**B**) Inhibition activity of the anti-ADAM17 mAb (100 ng/mL) against sRANKL release. *p < 0.05 between the groups.

To further confirm the neutralizing activity of anti ADAM17 mAb against ADAM17 expressed on cell surface, we examined the release of TNF-α and sRANKL from stimulated T-lymphocyte cell lines (EL4-TK) because ADAM17 cleaves TNF-α^35^ and sRANKL^36^,^37^. PMA/Ionomycin stimulation induced cleavage of both TNF-α and sRANKL (Fig. 2A,B). Anti ADAM17 neutralizing antibody inhibited the cleavage of both TNF-α and sRANKL. TAPI2 also inhibited both TNF-α and sRANKL cleavage (data not shown). These data suggested that anti ADAM17 neutralizing antibody efficiently inhibits the enzymatic activity of ADAM17.

### ADAM17 decreased concentration of IFN-γ in splenocyte cultures

**Figure 3:**
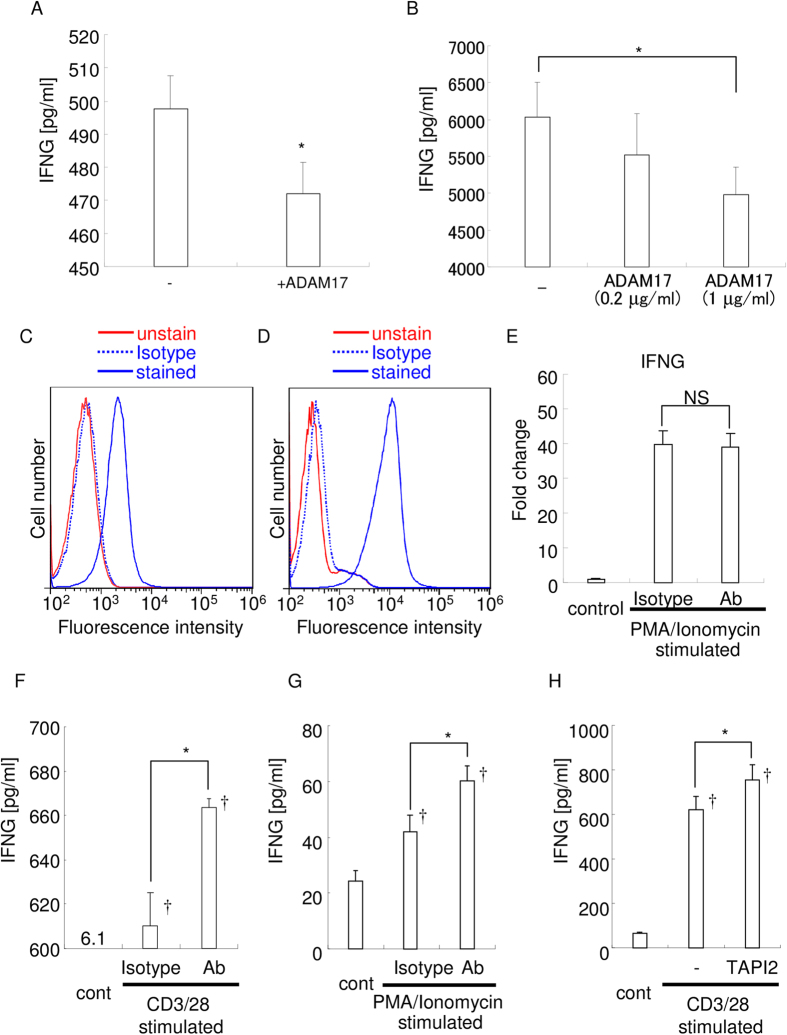
Addition of ADAM17 decreases the concentration of IFN-γ and vice versa. (**A**) Recombinant IFN-γ (500 pg/ml) was incubated with or without rADAM17 for 2 h, and IFN-γ concentration was measured by ELISA. −, without ADAM17; +ADAM17, with ADAM17; *p < 0.05. (**B**) Supernatants of PMA/Ionomycin-stimulated mouse splenocytes were incubated with rADAM17 for 2 h, and IFN-γ concentrations were measured by ELISA. *p < 0.05 between the groups. The expression of ADAM17 by CD3/28-stimulated mouse splenocytes (**C**) and PMA/Ionomycin-stimulated EL4-TK cells (**D**) was examined by flow cytometry. The red line indicates fluorescence of unstained cells, and the blue line shows fluorescence of cells stained with the CF647-conjugated anti-ADAM17 antibody. Blue dotted line showed the fluorescence of cells stained with the CF647-conjugated isotype IgG. The results are representative of three independent experiments. (**E**) Real-time PCR analysis of IFN-γ expression. Results are representative of three independent experiments. NS: no statistical difference between the groups. (**F**) Concentration of the intact IFN-γ in the culture supernatant of CD3/28-stimulated mouse splenocytes. *p < 0.05 between the groups; ^†^p < 0.05 versus control. (**G**) Concentration of intact IFN-γ in the culture supernatant of PMA/Ionomycin-stimulated EL4-TK cells. *p < 0.05 between the groups; ^†^p < 0.05 versus control. (**H**) Concentration of the intact IFN-γ in the culture supernatant of CD3/28-stimulated mouse splenocytes. *p < 0.05 between the groups; ^†^p < 0.05 versus control.

We then examined the effect of the rADAM17 on the concentration of recombinant IFN-γ, as measured by ELISA, and found that the addition of ADAM17 significantly reduced the concentration of IFN-γ (Fig. 3A). These data suggest that ADAM17 degrades recombinant IFN-γ into fragments that are too small for measurement by ELISA, in contrast to intact IFN-γ, which could be measured.

Next, we examined whether rADAM17 could degrade splenocyte-derived IFN-γ. We used culture supernatants of PMA/Ionomycin-stimulated mouse splenocytes, which contained a high concentration of native IFN-γ (6,026 pg/ml; Fig. 3B). It was found that the addition of rADAM17 reduced the concentration of splenocyte-derived IFN-γ in a dose-dependent manner (5,522 and 4,984 pg/ml, respectively). These data suggest that ADAM17 can degrade native IFN-γ and that ELISA could measure intact but not fragmented IFN-γ even in the presence of other factors, such as cell culture supernatant.

### ADAM17 inhibition increased IFN-γ in culture supernatants of ADAM17-expressing activated T cells

To further examine whether ADAM17 plays a role in IFN-γ degradation, we observed whether ADAM17 expressed by immune cells could degrade IFN-γ by using ADAM17-expressing activated T cells. Mouse primary T cells obtained from splenocytes and EL4-TK lymphoma T cells were stimulated with CD3/28 antibodies and PMA/Ionomycin, respectively. Flow cytometry revealed that both stimulated splenocytes and EL4-TK cells expressed ADAM17 (Fig. 3C,D). Then we analyzed the effect of the anti-ADAM17 neutralizing mAb on IFN-γ degradation. Real-time PCR analysis revealed that the addition of anti-ADAM17 neutralizing mAb did not show any statistical difference in IFN-γ mRNA expression by the cells stimulated with CD3/28 or PMA/Ionomycin (Fig. 3E).

ADAM17 inhibition by neutralizing antibody resulted in an increased concentration of IFN-γ in the culture supernatant of both stimulated splenocytes and EL4-TK cells (Fig. 3F,G). An ADAM family inhibitor, TAPI2, also increased IFN-γ concentration in CD3/28 stimulated splenocytes (Fig. 3H). These data suggest that the blockade of ADAM17 increased IFN-γ via the attenuation of degradation after secretion and not by transcriptional regulation. In addition, ADAM17 expressed by immune cells on cell surface could degrade native IFN-γ.

### Human breast cancer cell lines, MCF-7 and MDA-MB-453 expressed ADAM17 that degrades recombinant IFN-γ

**Figure 4:**
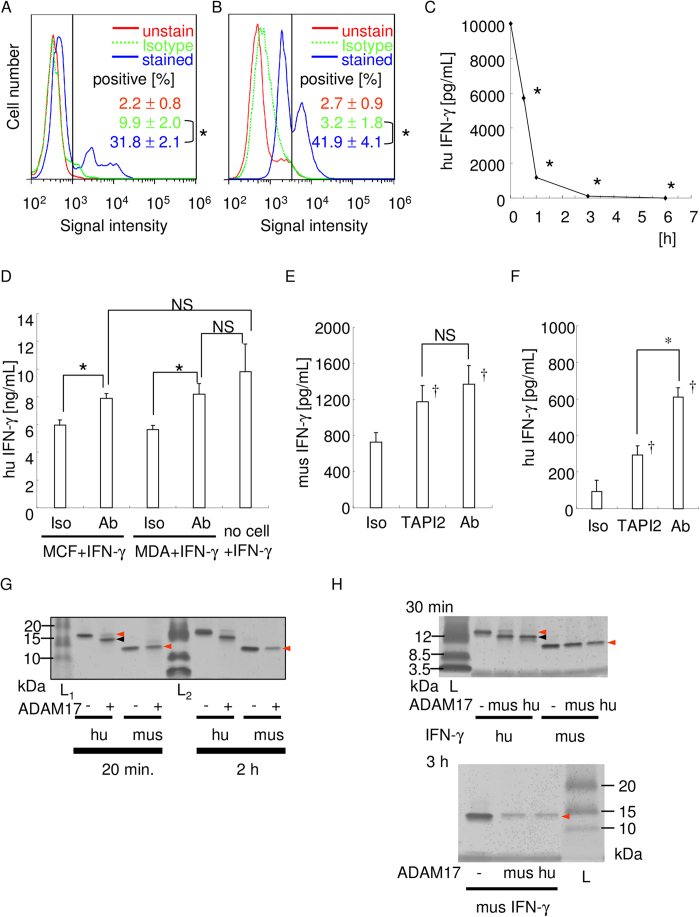
ADAM17-expressing breast cancer cells degrade IFN-γ in an ADAM17-dependent manner. The expression of ADAM17 by MCF7 (**A**) and MDA-MB-453 (**B**) cells were examined by flow cytometry. The red line indicates fluorescence of unstained cells, the dotted green line indicates fluorescence of CF647-conjugated isotype IgG stained cells, and the blue line shows fluorescence of stained cells, respectively. The results are representative of three independent experiments, and the mean percentage of ADAM17-positive cells is also shown. The vertical black line indicates the threshold. *p < 0.05 between the groups. (**C**) Time-course degradation of IFN-γ by MCF7 cells. IFN-γ concentration at each time point was measured. The representative result of three independent experiments is shown. *p < 0.05 versus 0 h. (**D–F**) ADAM17 blockade inhibited IFN-γ degradation. by MCF7 and MDA-MB-453 cells at 0.5 h. Recombinant human IFN-γ degradation by MCF7 and MDA-MB-453 cells at 0.5 h were shown (D). Recombinant mouse (E) or human (F) IFN-γ degradation by MCF7 cells at 3 h were shown. In the cell culture, isotype control IgG, anti-ADAM17 neutralizing antibody (100 ng/mL), or TAPI2 (10μM) was applied, respectively. *p < 0.05 between the groups. ^†^p < 0.05 versus isotype control IgG applied sample. NS: no statistically significant difference between the groups. (**G**) Comparison of ADAM17-mediated degradation of human and mouse IFN-γ. IFN-γ (200 ng) and ADAM17 (100 ng) was incubated for 20 min or 2 h, electrophoresed, and silver staining was performed. Red arrowhead indicates intact band, and the black arrowhead indicates degraded band. (**H**) Comparison of IFN-γ degradation activity of human and mouse ADAM17. Human or mouse ADAM17 (100 ng) was incubated with human or mouse IFN-γ (200 ng each) for 30 min or 3 h, electrophoresed, and silver staining was performed. Red arrowhead indicates intact band, and the black arrowhead indicates degraded band.

Based on the work of McGowan *et al*.^29^, as noted above, we analyzed whether breast cancer cells would exhibit ADAM17-dependent IFN-γ degradation. First, we observed the expression of ADAM-17 in MCF-7 and MDA-MB-453 cells. Flow cytometry analysis revealed that these cells do express ADAM17 (Fig. 4A,B). To investigate whether ADAM17-expressing MCF-7 cells could degrade IFN-γ, recombinant IFN-γ was added into MCF-7 culture, and the concentration in culture supernatant was measured by ELISA. Figure 4C shows a time-course decrease of IFN-γ concentration in the culture supernatant. Under the experimental conditions we used, IFN-γ was almost completely degraded within 3 h. Then, to determine if this IFN-γ degradation was ADAM17-dependent, anti-ADAM17 neutralizing mAb was applied into the culture system. As shown in Fig. 4D, IFN-γ concentration of the group that received neutralizing antibody was higher than the group that received no neutralizing antibody. Interestingly, no significant difference was observed between the group of MCF7 or MDA-MB-453 cells+IFN-γ+antibody and the group of no cells+IFN-γ, signifying that the addition of anti-ADAM17 neutralizing mAb almost completely inhibited IFN-γ degradation by these cells. This inhibition of IFN-γ degradation was also observed by the ADAM17 inhibitor, TAPI2, even at the later time point (Fig. 4E,F). These data suggest that ADAM17-expressing breast cancer cells, MCF7 and MDA-MB-453, can degrade IFN-γ within a few hours, but the inhibition of enzymatic activity of ADAM17 could completely block ADAM17-dependent IFN-γ degradation.

As we used mouse and human IFN-γ in the experiments and found some difference in the intact IFN-γ concentration (Fig. 4E,F), the susceptibility of each IFN-γ to ADAM17 were compared (Fig. 4G). Human IFN-γ was degraded by recombinant human ADAM17 within 20 min though there was no significant difference of the band density in mouse IFN-γ at same time period. The band density in mouse IFN-γ was weakened at 2 h, signifying that human IFN-γ is more susceptible than that of a mouse. Additionally, we compared the susceptibility of each IFN-γ to ADAM17 using recombinant mouse and human ADAM17, and found that human IFN-γ is more susceptible than that of a mouse irrespective of species of recombinant ADAM17 (Fig. 4H).

### ADAM17 expressed on MCF-7 dose dependently degrades recombinant IFN-γ

**Figure 5:**
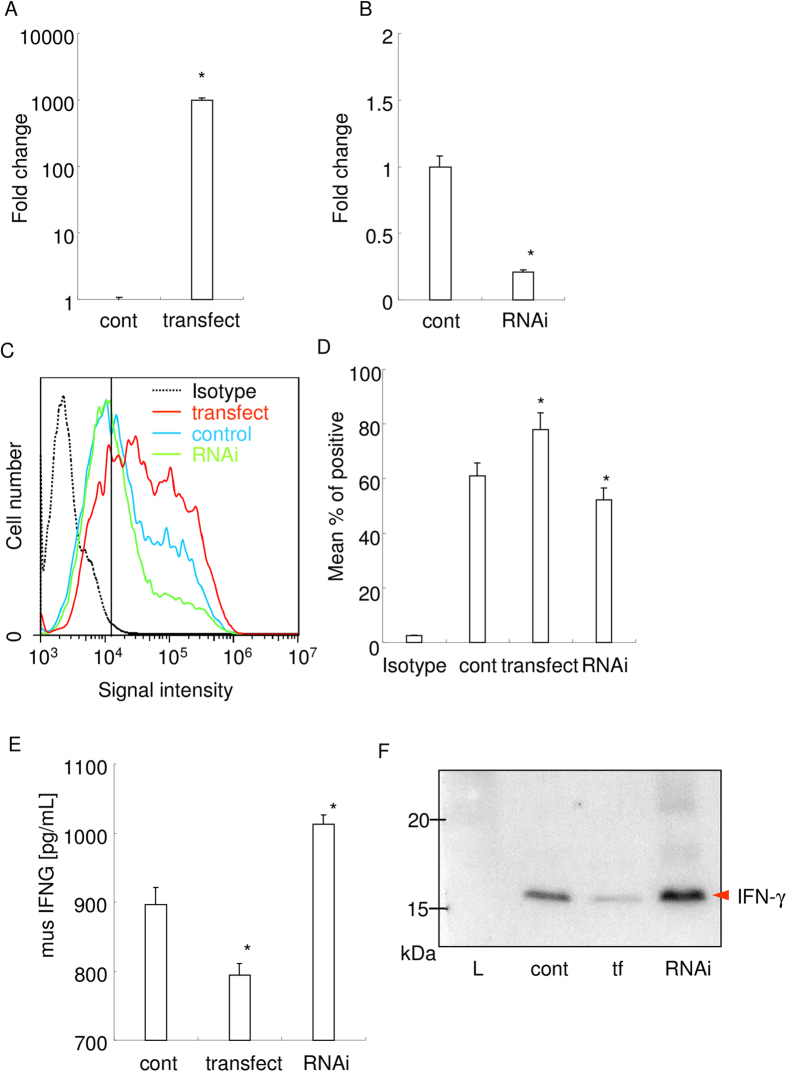
ADAM17 expressed on MCF-7 dose dependently degrades recombinant IFN-γ. (**A,B**) Real-time PCR analysis of ADAM17 mRNA expression in overexpressed or knocked down MCF-7. Fold changes from control were shown. *p < 0.05 versus control. ©Protein level expression of ADAM17 on the cell surface of MCF-7. Fc receptor blocking solution (Biolegend, San Diego, CA) was used prior to antibody application. The results of flow cytometry analysis are shown. Black dotted line indicates the result of fluorophore-conjugated isotype IgG. Blue, red, and green lines indicate the result of control cells, overexpressed cells, and knocked down cells, respectively. (**D**) Mean percent of positive cells from the results of flowcytometry analysis are shown. *p < 0.05 versus control. (**E**) IFN-γ concentration measured by ELISA. 1 ng/mL of recombinant mouse IFN-γ were added into each culture media, and incubated for 1 hour. *p < 0.05 versus control. (**F**) Western blot analysis of IFN-γ performed using the collected culture supernatant. tf: overexpressed cells. RNAi: knocked down cells. L: molecular weight marker.

Next, we overexpressed or knocked down the ADAM17 expression on MCF-7 whether IFN-γ concentration shows negative correlation to the ADAM17 expression. Realtime PCR analysis revealed that the successful overexpression and knockdown of ADAM17 mRNA expression in MCF-7 (Fig. 5A,B). The flow cytometry analysis also confirmed the protein level overexpression and knockdown of ADAM17 on the MCF-7 cell surface (Fig. 5C,D). As compared to the control, ADAM17 overexpression reduced IFN-γ concentration (Fig. 5E). On the other hand, ADAM17 knockdown increased IFN-γ concentration. These changes in IFN-γ concentration in the culture supernatant were further confirmed by western blot analysis of the supernatant (Fig. 5F). These results suggest that ADAM17 expressed on the cell surface of MCF-7 dose dependently degraded IFN-γ.

### IFN-γ degradation attenuates inhibitory effect of IFN-γ on osteoclastogenesis

**Figure 6:**
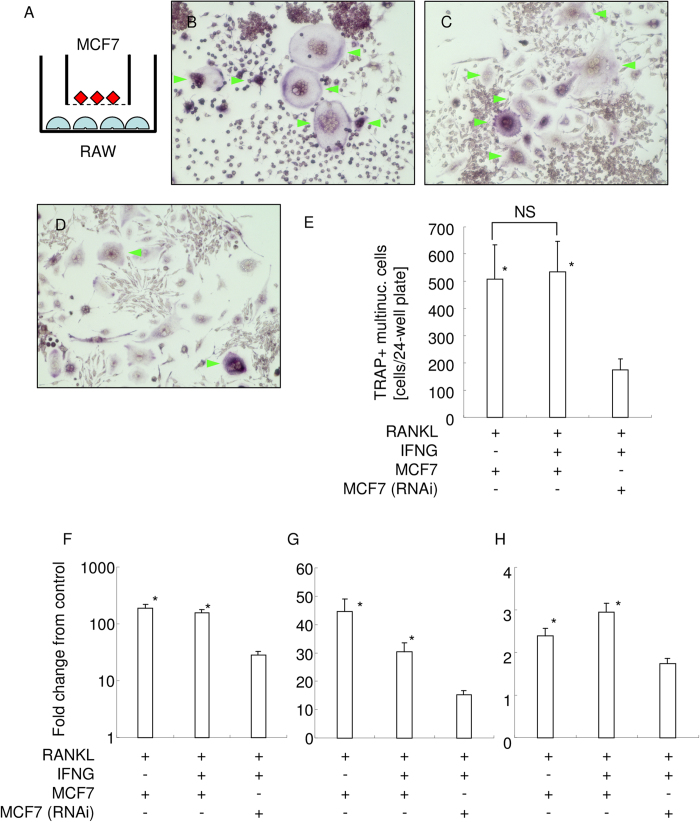
Biological function analysis of ADAM17-mediated IFN-γ degradation. (**A**) Schematic illustration of indirect coculture between RAW 264.7 cells and MCF7 cells. ϕ 1μm cell culture filter insert was used. TRAP staining of RAW 264.7 cells coculture with MCF7 cells in the presence of RANKL (**B**), coculture with MCF7 cells in the presence of RANKL and IFN-γ (**C**), and coculture with ADAM17-knockeddown MCF7 cells in the presence of RANKL and IFN-γ (**D**) are shown, respectively. Green arrowhead indicates TRAP+ multinucleated cells. (**E**) The number of TRAP+ multinucleated cells in each group are shown. *p < 0.05 vs coculture with ADAM17-knockeddown MCF7 cells in the presence of RANKL and IFN-γ. NS: no significant difference between the groups. Real-time PCR analysis for osteoclast marker gene expression. Fold change from the control of TRAP (**F**), ATP6v0d2 (**G**), and cathepsin K (**H**) were shown, respectively. *p < 0.05 VS coculture with ADAM17-knockeddown MCF7 cells in the presence of RANKL and IFN-γ.

Finally, we examined biological function of ADAM-17-mediated IFN-γ degradation using indirect coculture between RAW 264.7 cells and MCF7 cells (Fig. 6A). Indirect coculture with MCF7 cells had no inhibitory effect on RANKL-mediated osteoclastogenesis (Fig. 6B). Though several reports indicated an inhibitory effect of IFN-γ on RANKL-mediated osteoclastogenesis, the indirect coculture with MCF7 cells in the presence of IFN-γ formed TRAP+ multinucleated cells (Fig. 6C). There was no statistically significant difference in the number of TRAP+ multinucleated cells (Fig. 6E). However, indirect coculture with ADAM17-knockeddown MCF7 cells in the presence of IFN-γ formed much less TRAP+ multinucleated cells (Fig. 6D). There is a statistically significant difference with the number of TRAP+ multinucleated cells (Fig. 6E).

To further clarify the effects of ADAM17-mediated IFN-γ degradation on osteoclastogenesis, we examined the expression of osteoclast marker genes, TRAP (Fig. 6F), ATP6v0d2 (Fig. 6G), and cathepsin K (Fig. 6H). Indirect coculture with ADAM17-knockeddown MCF7 cells in the presence of IFN-γ gave lower expression of marker genes, signifying inhibition of osteoclastogenesis in this condition.

These results suggest that ADAM17-mediated IFN-γ degradation attenuates the biological functional activity of IFN-γ.

## Discussion

In the present study, it was found that ADAM17 proteinase enzymatically degrades and inactivates IFN-γ. This activity proved to be specific because neither heat-inactivated ADAM17 nor ADAM10 could degrade IFN-γ. Furthermore, our experiments involving either the addition of the exogenous rADAM17 to culture supernatants, or the inhibition of ADAM17 by a neutralizing antibody, clearly demonstrate that the negative correlation of IFN-γ concentration with ADAM17 activity. Taken together, the results suggest the possibility of degradation of the ADAM17-expressing cancer cells, which may result in the inactivation of IFN-γ. Indeed, ADAM17-expressing breast cancer cell-lines MCF-7 and MDA-MB-453 degrade IFN-γ within only a few hours. Our overexpression and knockdown experiments of ADAM17 in MCF-7 cells clearly demonstrated that cell surface ADAM17 dose dependently degrades IFN-γ.

Since IFN-γ can promote anti-tumorigenic effects through the activation of natural killer cell surveillance^1^,^2^,^3^,^4^, it is thought to be a prognostic factor for cancer therapy^5^,^6^,^7^,^8^. IFN-γ is also known as an anti-osteoclastogenic cytokine by its degradation of TRAF6, which results in the subsequent inhibition of RANKL signaling in osteoclasts^9^. During bone cancer metastasis, RANKL plays an important role in the activation of osteoclastic bone resorption^38^,^39^, and the anti-RANKL therapy for treatment and prevention of bone cancer metastasis is currently under development^40^. Thus, the inhibition of RANKL signaling by IFN-γ can be a promising approach toward the prevention of osteoclastic bone resorption, and the prospects of using IFN-γ to treat bone cancer metastasis have already been reported^41^. Since the expression of ADAM17 was found to correlate with tumor progression in breast cancer^29^, it was further hypothesized that ADAM17 produced by cancer cells might degrade IFN-γ in tumorigenic sites and thus attenuate IFN-γ-dependent cancer surveillance and anti-osteoclastogenic activity. Indeed, our results of western blot analysis for TRAF6 clearly indicated that degraded IFN-γ could not inhibit TRAF6 signaling, signifying the functionality loss of the degraded IFN-γ fragments. Furthermore, the coculture experiment clearly demonstrated that ADAM17-mediated IFN-γ degradation attenuated the biological function of IFN-γ.

While the results of the present study confirmed that ADAM17 degrades IFN-γ, the IFN-γ cleavage motif recognized by ADAM17 has not yet been identified. According to the peptidase database (MEROPS: http://merops.sanger.ac.uk/index.shtml)^42^, the cleavage site specificity of ADAM17 is not as strict as that of other peptidases. Indeed, ADAM17 cleaves a variety of proteins, including tumor necrosis factor (TNF)-α^35^, RANKL^36^, transforming growth factor (TGF)-α^43^, amyloid precursor protein^44^, amphiregulin^45^, and chemokine (C-X-C motif) ligand (CXCL) 16^46^. Cleavage site consensus of ADAM17 shows a preference for the P1′ position, immediately downstream of the cleavage site of the substrate. Here, it is selective for smaller aliphatic residues, including valine, leucine, or serine [48], and, notably, IFN-γ, which contains a similar sequence. As we found that human IFN-γ is more susceptible than the mouse IFN-γ, these results would give a clue for identifying the cleavage site. Homology search resulted in 66% positive match between human and mouse IFN-γ (Supplementary Figure 2), signifying that the difference in the sequence might result in the difference of susceptibility. N-terminal sequencing of the digested small IFN-γ fragment is necessary to identify the exact cleavage site for ADAM17 in IFN-γ.

We observed that the ADAM17-expressing breast cancer cell lines MCF-7 and MDA-MB-453 cells could degrade IFN-γ within only a few hours, even though IFN-γ is known as a cytokine that can promote anti-tumorigenic effects through the activation of NK cell surveillance^1^,^2^,^3^,^4^. To explain the significance of this suppressive effect, we turn to Engel *et al*.^47^ who reported that NK cell-induced tumor cell lysis was significantly more pronounced in triple negative breast cancer cells, where estrogen receptor (−) and progesterone receptor (−) did not over-express the HER-2 receptor, compared to ER- positive MCF7 cells^47^, indicating that MCF7 cells are prone to resist NK cell cancer surveillance. Taken together, it therefore seems highly possible that MCF7 cells escape cancer surveillance via attenuation of NK cells upon the degradation of IFN-γ. Since our results of overexpression experiments for ADAM17 in MCF7 cells indicated the dose-dependency between ADAM17 expression and IFN-γ degradation, i.e., IFN-γ inactivation, our results may explain the correlation of tumor progression in breast cancer to the extent of ADAM17 expression^29^. Importantly, however, this also means that ADAM17 could be a therapeutic target molecule for the inhibition of escape from cancer surveillance.

To the best of our knowledge, this is the first study to report on the modifications of secreted IFN-γ. Our findings have shed light on the association between cancer immunosurveillance and enzyme activity profiles of cancer cells. More importantly, we have demonstrated that the inhibition of ADAM17 proteolytic activity with a neutralizing antibody could prevent IFN-γ degradation, strongly suggesting that such inhibition could be utilized as a therapeutic approach to enforce cancer immune surveillance and inhibit osteoclastic bone resorption during bone cancer metastasis.

## Methods

### Animals

All the experimental protocols were approved by the Internal Animal Care and Use Committee, Tohoku University. Furthermore, the animal experiments were performed in compliance with the Regulations for Animal Experiments and Related Activities at Tohoku University.

### Primary cells and cell lines

Mouse splenocytes were obtained from BALB/c mice (Japan SLC, Hamamatsu, Japan), after sacrifice. Spleens were excised, minced, and splenocytes were collected and used for *in vitro* experiments and for preparation of the hybridoma. Mouse myeloma SP-2 cell-line and IL-2-producing mouse T lymphoma EL4-TK cell-line were obtained from the Cell Resource Center for Biomedical Research, Institute of Development, Aging and Cancer, Tohoku University (Sendai, Japan). Human breast cancer MCF-7 cell line, MDA-MB-453 cell line and mouse macrophage cell line RAW 264.7 cells were obtained from Riken Bioresource Center cell bank (Tsukuba, Japan).

### Cell culture

Cells were cultured in alpha-modified Eagle’s Medium (Wako Pure Chemical Industries, Osaka, Japan) containing 10% fetal bovine serum supplemented with penicillin and streptomycin.

### IFN-γ degradation assay

Recombinant IFN-γ (1 μg; Wako) was incubated with intact or heat-inactivated recombinant ADAM17 (rADAM17) or ADAM10 (100 ng each; R&D Systems, Minneapolis, MN) in assay buffer (25 mM Tris, 2.5 μM ZnCl_2_, 0.005% Brij-35, pH 9.0) for 1.5 h. Then NuPAGE LDS Sample Buffer (Life Technologies, Tokyo, Japan) containing reducing agent was added to each sample. After boiling, the protein samples were separated by Mini-PROTEAN Tris-Tricine gel (Bio-Rad Laboratories, Hercules, CA), electrophoresis, and stained with Bio-Safe Coomassie Brilliant Blue (Bio-Rad) or Silver Stain MS Kit (Wako). In some experiments, a protein band was excised and subjected to nano LC-MS/MS analysis for peptide sequence identification (Japan Proteomics, Sendai, Japan).

To observe the degradation activity of ADAM17 against other IFNs, recombinant human interferon β1a (ProSpec-Tany TechnoGene, Rehovot, Israel) was tested as a substrate.

### Preparation of the human ADAM17 immunogenic peptides

To generate an anti-ADAM17 neutralizing antibody, we chose nine peptide sequences (<25 amino acids) within the extracellular domain, and synthesized (Life Technologies Japan). Peptide sequences/positions are shown in Supplementary Table 1.

### Immunization of mice with ADAM17 synthetic peptides

Synthesized ADAM17 peptides were conjugated with Keyhole Limpet Hemocyanin (KLH; Wako) by using disuccinimidyl suberate (DSS; Thermo Scientific, Rockford, IL). KLH-conjugated ADAM17 peptides were emulsified with TiterMax Gold adjuvant (CytRx, Norcross, GA). Eighteen BALB/c mice were randomly divided into nine groups (n = 2 each), and the emulsions were immunized every other week.

### Preparation of polyclonal anti-ADAM17 peptide antibodies

Three days after the final injection, the mice were sacrificed, and blood and spleens were collected. Sera were separated by centrifugation, and IgG was purified using Ab-Rapid spin column (ProteNova, Tokushima, Japan). All ADAM17 peptides were conjugated with NHS-Activated Agarose (Thermo Scientific), and polyclonal anti-peptide antibodies were purified. Purified antibodies were buffer-exchanged into PBS, and their concentrations were adjusted equally.

### Blocking of ADAM17 enzymatic activity by antibodies, as measured by fluorescence intensity

An equal amount of each antibody (10 ng) was pre-incubated with recombinant ADAM17 (12.5 ng), followed by the addition of ADAM17 fluorogenic peptide substrate (R&D Systems). In some experiments, an ADAM family inhibitor, TAPI2 (Santa Cruz Biotechnology, Santa Cruz, CA^48^), was used as a control. Fluorescence (excitation: 320 nm, emission: 405 nm) was measured with fluorescence plate reader, and assessed ADAM17 enzymatic activity was assessed.

### Generation of an anti-ADAM17 peptide antibody-producing hybridoma

Splenocytes from immunized mice were fused with parental myeloma cells. Then these fused hybridoma cells were cultured with ClonaCell-HY (Stemcell Technologies, Vancouver, BC), and colonies were obtained. The colonies were positively selected according to the reactivity of produced IgG with the ADAM17 peptide and rADAM17, and then negatively selected according to their reactivity with the carrier protein KLH. Neutralizing activity against rADAM17 was examined as described above.

### Epitope mapping of the monoclonal antibody

Peptides for epitope mapping of the monoclonal antibody (mAb) against peptide No. 9 (VMYPIAVSGDHENNKMFSNCSKQ) were synthesized (Medical & Biological Laboratories, Nagoya, Japan). The sequences are presented in Supplementary Table 2. The mAb epitopes were examined using the following two methods:

1. Inhibition of the antibody binding to rADAM17: ELISA plates were coated with rADAM17 and blocked. The mAb was pre-incubated with each epitope-mapping peptide and then applied to ADAM17-coated ELISA plates. After washing, the bound antibody was detected using horseradish peroxidase (HRP)-conjugated anti-mouse IgG, followed by tetramethylbenzidine (TMB) solution (Sigma, St. Louis, MO).
2. Binding of the antibody to epitope-mapping peptides: Epitope-mapping peptides were immobilized on a high-binding ELISA plate and blocked. The mAb was applied into each well, washed, and the bound antibody was detected as described above.

### IFN-γ degradation assay using the native protein

Mouse splenocytes were stimulated with anti-CD3/CD28 antibodies-coated beads (DynaBeads; Life Technologies Japan), and EL4-TK cells were stimulated with PMA and Ionomycin for 24 h, and culture medium was exchanged. Then the cells were further cultured with anti-ADAM17 mAb or isotype IgG (normal mouse IgG) for 24 h, and the culture supernatants were collected. IFN-γ concentrations in the culture supernatant were measured by ELISA (BioLegend, San Diego, CA).

To confirm that the native IFN-γ produced by mouse splenocytes could be degraded by rADAM17, culture supernatants of PMA/Ionomycin-stimulated mouse splenocytes were incubated with the rADAM17 for 2 h, and the IFN-γ concentrations were measured.

### IFN-γ degradation assay using the recombinant protein

To observe whether MCF-7 cells could degrade IFN-γ, recombinant IFN-γ (final: 10 ng/mL) was added to MCF-7 cells (2 × 10^5^/well, 24-well plate). After 30 min, the culture supernatants were collected and examined for the concentration of IFN-γ by original ELISA, as described below. To confirm if IFN-γ degradation was dependent on ADAM17, neutralizing anti-ADAM17 mAb or isotype IgG (normal mouse IgG) was added 5 min prior to IFN-γ addition, and the culture supernatants were collected.

### Human IFN-γ ELISA

Briefly, the plate was coated with capture antibody (rabbit polyclonal IgG, anti- IFN-γ antibody; SC-8308, Santa Cruz) and blocked. After incubation of samples and standards (from 10 ng/mL to zero), the detection antibody (goat polyclonal IgG, anti-IFN-γ antibody; SC-1377, Santa Cruz) was applied and washed, and the HRP-conjugated anti-goat IgG secondary antibody was incubated. After washing, TMB solution was applied, and the absorbance was measured at 630 nm. In this system, sensitivity, i.e., concentration of analyte giving absorbance higher than the mean absorbance of blank plus three standard deviations of the absorbance of blank, was found to be 84.7 pg/mL.

### ADAM17 detection by flow cytometry

ADAM17 expression by CD3/28 antibody-stimulated mouse splenocytes, PMA/Ionomycin-stimulated EL4-TK cells, MCF-7, and MDA-MB-453 cells were examined by flow cytometry. Briefly, the harvested cells were incubated with rat IgG anti-ADAM17 polyclonal antibodies^49^ conjugated with fluorophore CF647 (Biotium, Hayward, CA) or isotype IgG (normal rat IgG)-CF647, and washed. Fluorescence was detected using an AccuriC6 flow cytometer (BD Biosciences, Franklin Lakes, NJ). The viable cell fraction was gated on an FSC/SSC plot for each cell type, and the fluorescence of CF647 was monitored in the FL-4 channel.

### Real-time RT-PCR analysis for IFN-γ expression

RNA was extracted from the cultured cells using the GenElute Mammalian Total RNA Miniprep Kit (Sigma). On-column DNase treatment was performed to digest genomic DNA during RNA extraction. Isolated RNA (100 ng each) was reverse transcribed with iScript cDNA Supermix (Bio-Rad). Real-time RT-PCR was performed with SsoFast EvaGreen Supermix (Bio-Rad) using a CFX96 instrument (Bio-Rad). The PCR primers used in the experiments were from PrimerBank (http://pga.mgh.harvard.edu/primerbank/index.html). We used ribosomal protein S18 (RPS18) as the reference gene. The PrimerBank ID and sequences are as follows: IFN-γ (NM_008337.3):33468859a1, Up:ATGAACGCTACACACTGCATC; Dn:CCATCCTTTTGCCAGTTCCTC and RPS18 (NM_011296):6755368a1, Up:AGTTCCAGCACATTTTGCGAG; Dn:TCATCCTCCGTGAGTTCTCCA.

### Overexpression and knockdown of ADAM17

The expression plasmid for human ADAM17 (Origine, Rockville, MD) was transfected into MCF7 cells. siRNA for human ADAM17 (AUGAGUUGUAACCAGGUCAGCUUCC) was synthesized (Eurofins Genomics, Tokyo, Japan) and transfected into MCF7 cells. The details of transfection have been previously described elsewhere^50^. Overexpression and knockdown efficiency at 24 h were monitored by real-time RT-PCR and flowcytometry. Then 2 ng of recombinant IFN-γ was applied into 2 mL culture media, and incubated for half hour. The concentration of IFN-γ in the culture supernatant was measured by ELISA, and further examined using western blot analysis (primary antibody: sc-8308).

### Functional assay of degraded IFN-γ

Degraded IFN-γ was separated from intact IFN-γ using 10kDa MWCO spin column (EMD Millipore, Billerica, MA) and concentrated using 3kDa MWCO spin column. Then the concentration of separated degraded IFN-γ fragment was measured using Qubit protein assay kit and Qubit 3.0 fluorometer (Invitrogen), and calibrated into the same concentration (7.1 ng/μL). RAW 264.7 cells were stimulated by sRANKL (100 ng/mL; Wako) with intact or degraded IFN-γ fragment (7.1 ng/mL each) or without IFN-γ for 2 days. The whole cell lysate was prepared using lysis buffer (5mM EDTA, 10% glycerol, 1% triton X-100, 0.1% SDS, 1% NP-40 in PBS) containing proteinase inhibitor cocktail (Wako). The protein concentration was measured and calibrated into equal amounts, which were then electrophoresed into TGX Precast gel (BioRad), and transferred to a polyvinylidene difluoride membrane. The membrane was blocked, and then incubated for 1 h with rabbit IgG anti-TRAF6 antibody (Bioworld Technology, St. Louis Park, MN). After washing, the membrane was incubated for 1 h with HRP-conjugated protein A/G, and washed. Chemiluminescence was produced using Luminata Forte (EMD Millipore, Billerica, MA) and detected with LumiCube (Liponics, Tokyo, Japan).

### Statistical analysis

All data are presented as mean ± standard deviation (SD) of three independent experiments. Multiple comparisons were performed with Tukey’s test (http://www.gen-info.osaka-u.ac.jp/testdocs/tomocom/tukey-e.html). P < 0.05 was considered statistically significant.

## Additional Information

**How to cite this article**: Kanzaki, H. *et al*. A-Disintegrin and Metalloproteinase (ADAM) 17 Enzymatically Degrades Interferon-gamma. *Sci. Rep*. **6**, 32259; doi: 10.1038/srep32259 (2016).

## Supplementary Material

Supplementary Information
