# Supplementary material for: A-Disintegrin and Metalloproteinase (ADAM) 17 Enzymatically Degrades Interferon-gamma
Source: Sci Rep. 2016 Aug 30;6:32259. doi: 10.1038/srep32259 (PMC5004192; doi:10.1038/srep32259)
Supplement: Supplementary Information [file srep32259-s1.pdf]

Supplementary tables

Title: A-Disintegrin and Metalloproteinase (ADAM) 17 Enzymatically Degrades Interferon-gamma

Authors: Hiroyuki Kanzaki[1,2*], Fumiaki Shinohara[3], Maiko Suzuki[4,5], Satoshi Wada[1], Yutaka Miyamoto[1], Yuuki Yamaguchi[1], Yuta Katsumata[1], Seicho Makihira[8], Toshi Kawai[4,6], Martin A. Taubman[4,7], and Yoshiki Nakamura[1]

Supplementary table 1. Immunogen of human ADAM17

Peptide No.	Amino Acid Sequence	Position
1	HRFYRYMGRGEESTTTNY	233 - 250
2	DIYRNTSWDNAGFKG	260 - 274
3	IRILKSPQEVKPGEKH	282 - 297
4	MAKSYPNEEKDAW	300 - 312
5	DFDMGTLGLAYVGSPRANSHG	342 - 362
6	KAYYSPVGKKNIY	367 - 379
7	KNYGKTILTKEADLVTTHELGHNFG	388 - 412

8	EHDPDGLAECAPNEDQGGKY	414 - 433
9	VMYPIAVSGDHENNKMFSNCSKQ	434 - 456

Human ADAM17: UniProt P78536

Extracellular domain: 215 - 671

Peptidase domain: 223 - 474

Active site: 406

Supplementary table2. Peptide sequences for epitope mapping

Peptide	Sequence
9A	VMYPIAVS
9B	YPIAVSGD
9C	IAVSGDHE
9D	VSGDHENN
9E	GDHENNKM
9F	HENNKMFS
9G	NNKMFSNC
9H	KMFSNCSKQ

Figure legends for Supplementary figures

Title: A-Disintegrin and Metalloproteinase (ADAM) 17 Enzymatically Degrades Interferon-gamma

Authors: Hiroyuki Kanzaki[1,2*], Fumiaki Shinohara[3], Maiko Suzuki[4,5], Satoshi Wada[1], Yutaka Miyamoto[1], Yuuki Yamaguchi[1], Yuta Katsumata[1], Seicho Makihira[8], Toshi Kawai[4,6], Martin A. Taubman[4,7], and Yoshiki Nakamura[1]

Supplementary figure 1 Characteristics of established anti ADAM17 monoclonal antibody.

A: Nine potential epitopes (sequences are shown in Table1) were selected, and corresponding synthetic peptides were used for immunization. Polyclonal antibodies were compared by the blocking activity towards ADAM17 and presented as a percentage of ADAM17 activity in control samples (100%). Higher values indicate weaker inhibition activity of an antibody.

B: The epitope sequence of the obtained mAb determined by epitope mapping using eight peptides (Table 2). Inhibition of antibody binding to rADAM17 by the antibody pre-incubated with or without peptides was examined, and binding of

the anti-ADAM17 mAb is shown. Lower values indicate stronger binding of a peptide to the mAb. 9A-9H, pre-incubation of the antibody with peptides 9A to 9H, respectively; −, antibody not pre-incubated with a peptide. * $p < 0.05$ to control.

C: Binding of the anti-ADAM17 mAb to epitope-mapping peptides. Higher values indicate stronger binding of the antibody to a peptide. 9A-9H, wells coated with peptides 9A to 9H, respectively; no coating = wells with buffer.

D: Inhibition activity of the anti-ADAM17 mAb and TAPI2. Four and five serial dilutions of the antibody and TAPI2, respectively, were used for pre-incubation with equal amounts of rADAM17. Activity was assessed using a fluorescent substrate and presented as a percentage of ADAM17 activity in control (100%).

Supplementary figure 2 Homology search of human and mouse IFN-γ.

Homology search of human IFN-γ (Uniprot P01579.1) and mouse IFN-γ (Uniprot P01580.1) were performed using Standard Protein BLAST (http://blast.ncbi.nlm.nih.gov/).

Supplementary Figure 1

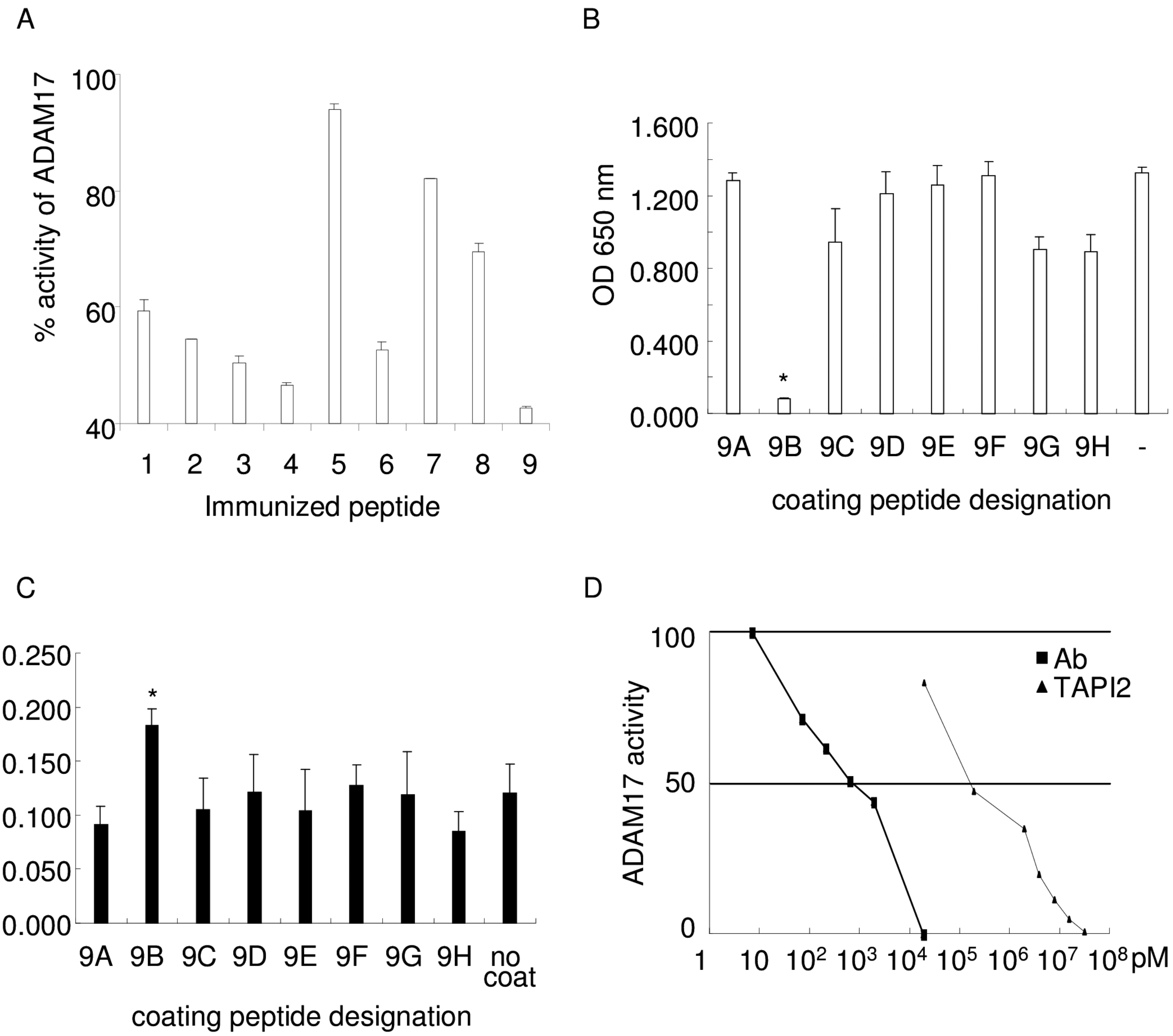

Supplementary figure 2

```
human    5    VKEAENLKKYFNAGHSDVADNGTLFLGILKNWKEESDRKIMQSQIVSFYFKLFKNFKDDQ  64
              ++  E+L  YFN+   DV +  +LFL I +NW+++ D KI+QSQI+SFY +LF+  KD+Q
mouse   27    IESLESLNNYFNSSGIDVEEK-SLFLDIWRNWQKDGDMKILQSQIISFYLRLFEVLKDNQ  85

human   65    SIQKSVETIKEDMNVKFFNSNKKKRDDFEKLTNYSVTDLNVQRKAIHELIQVMAELSPAA  124
              +I  ++  I+  +   FF+++K K+D F  +  + V +  VQR+A +ELI+V+ +LSP +
mouse   86    AISNNISVIESHLITTFFSNSKAKKDAFMSIAKFEVNNPQVQRQAFNELIRVVHQLSPES  145

human  125    KTGKRKRSQM  134
                 KRKRS+
mouse  146    SLRKRKRSRC  155
```

Identities	53/130 (41%)
Positives	87/130 (66%)
Gaps	1/130 (0%)
